# Supplementary material for: Chromothripsis during telomere crisis is independent of NHEJ, and consistent with a replicative origin
Source: Genome Res. 2019 May;29(5):737–49. doi: 10.1101/gr.240705.118 (PMC6499312; doi:10.1101/gr.240705.118)
Supplement: Supplemental Material [file supp_gr.240705.118_Supplemental_file_1.zip › contigs/annotated_contigs/DB116/contig.2.DB116_length_303_mean_cov_3.9603960396.docx]

**DB116_length_303_mean_cov_3.9603960396**

```
ATTCTTGGTTTTGTACTGTCCTAAATGGCAATGTGACTGAGGGCAAGTTATTTAAACCTTTTCATCACACACATCATTTGTAAAATATA
 >chr4:163087563-163087757 + E=1e-105 p=4e-02
TAGGTTATCCTGTTATCTCCAAAATTCATTACATGCTTGTCATTCTGTAATTTTATATTAGACTTCAACCTATTTTATATATATATATA

ATTTGGAATCAC|TATA|TATATGGCATCTTCTTTACTTCCATTACTTTCTTACTTTCTATCTCTTGCTCCCTTGGGCCCTTTTGTGCT
             >chr4:163087884-163087996 + E=4e-56
CTCCACATATGTTTTATTTCATTGTAATGTTCTATTT|T
```
